# *N*-Ethyl-*N*-phen­yl{[eth­yl(phen­yl)carbamothio­yl]disulfan­yl}carbothio­amide

**DOI:** 10.1107/S1600536812027808

**Published:** 2012-06-23

**Authors:** Peter A. Ajibade, Benjamin C. Ejelonu, Bernard Omondi

**Affiliations:** a Department of Chemistry, University of Fort Hare, Private Bag X1314, Alice 5700, South Africa; b School of Chemistry and Physics, University of KwaZulu-Natal, Westville Campus, Private Bag X54001, Durban, 4000, South Africa

## Abstract

The asymmetric unit of the title compound, C_18_H_20_N_2_S_4_, contains one half-mol­ecule, the complete molecule being generated by a twofold rotation axis. The plane through the NCS_2_ group [maximum deviation = 0.01 (7) Å] is orthogonal to the phenyl ring, forming a dihedral angle of 89.4 (3)°. The crystal structure is stabilized by inter­molecular C—H⋯π inter­actions.

## Related literature

For background to the chemistry of thiuram disulfides and their potential applications, see: Chieh (1977 ▶); McCleverty & Morrison (1976 ▶); Victoriano (2000 ▶). For related structures, see: Fun *et al.* (2001 ▶); Raya *et al.* (2005 ▶).
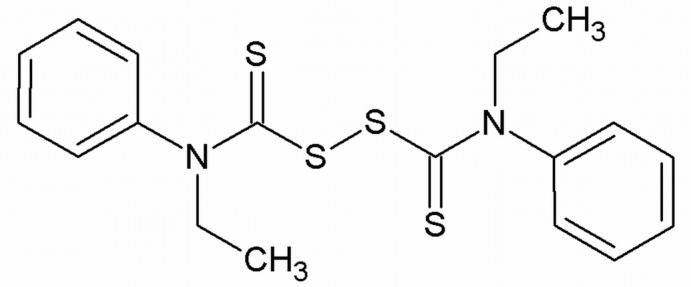


## Experimental

### 

#### Crystal data

C_18_H_20_N_2_S_4_
*M* *_r_* = 392.6
Monoclinic, 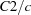

*a* = 15.1923 (2) Å
*b* = 11.5954 (2) Å
*c* = 12.3762 (2) Å
β = 115.039 (1)°
*V* = 1975.31 (5) Å^3^
*Z* = 4
Mo *K*α radiation
μ = 0.48 mm^−1^
*T* = 100 K
0.40 × 0.37 × 0.15 mm

#### Data collection

Bruker APEXII CCD diffractometer
Absorption correction: multi-scan (*SADABS*; Bruker, 2008 ▶) *T*_min_ = 0.830, *T*_max_ = 0.931
19720 measured reflections
2481 independent reflections
2405 reflections with *I* > 2σ(*I*)
*R*_int_ = 0.027

#### Refinement

*R*[*F* ^2^ > 2σ(*F* ^2^)] = 0.024
*wR*(*F* ^2^) = 0.068
*S* = 1.06
2481 reflections
110 parameters
H-atom parameters constrained
Δρ_max_ = 0.40 e Å^−3^
Δρ_min_ = −0.29 e Å^−3^

### 

Data collection: *APEX2* (Bruker, 2008 ▶); cell refinement: *SAINT-Plus* (Bruker, 2008 ▶); data reduction: *SAINT-Plus* and *XPREP* (Bruker, 2008 ▶); program(s) used to solve structure: *SHELXS97* (Sheldrick, 2008 ▶); program(s) used to refine structure: *SHELXL97* (Sheldrick, 2008 ▶); molecular graphics: *ORTEP-3* (Farrugia, 1997 ▶); software used to prepare material for publication: *WinGX* (Farrugia, 1999 ▶).

## Supplementary Material

Crystal structure: contains datablock(s) global, I. DOI: 10.1107/S1600536812027808/ru2038sup1.cif

Structure factors: contains datablock(s) I. DOI: 10.1107/S1600536812027808/ru2038Isup2.hkl

Additional supplementary materials:  crystallographic information; 3D view; checkCIF report

## Figures and Tables

**Table 1:** Hydrogen-bond geometry (Å, °) *Cg* is the centroid of the C1–C6 ring.

*D*—H⋯*A*	*D*—H	H⋯*A*	*D*⋯*A*	*D*—H⋯*A*
C8—H8*B*⋯*Cg* ^i^	0.98	2.97	3.7972 (14)	143

Symmetry code: (i) 
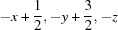
.

**Fig. 1.**
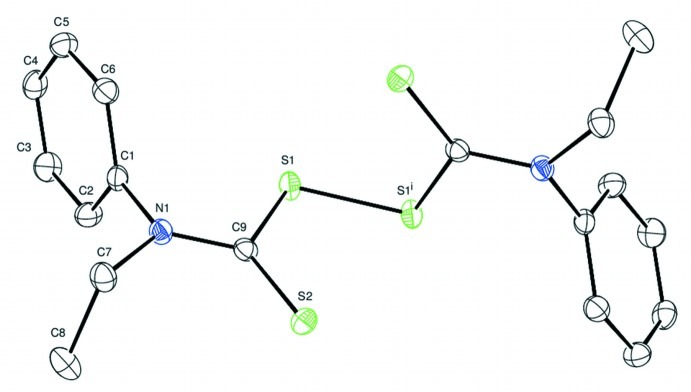
The asymmetric unit of the title compound with displacement ellipsoids drawn at the 50% probability level.

## supplementary crystallographic information

### Comment

Thiuram disulfides are semi-esters of dialkyldithiocarbamic acids (Victoriano, 2000). They are unique among thiolato type ligands in that reductive scission of the S—S bond leads to chelating dithiocarbamtes anions which are particularly well suited to stabilize high oxidation state transition metals like dithiocarbamate ligands (Victoriano, 2000). Metal species with closed shell configuration typically react with thiuram disulfides to yield adducts. Some adducts of well defined thiuram complexes have been obtained by the reaction of group 12 halides and the ligands McCleverty & Morrison (1976)).

The structure of (I) Fig. 1, consists of two *N*ethyl-*N*-phenyldithiocarbamate units linked by an S–S bond. The plane of NCS_2_ group is orthogonal to the plane of the phenyl ring forming a dihedral angle of 89.40 (3)° between them. The torsion angle between the thiocarbamate moieties (NCS_2_) is 79.01 (8)°. The lattice is stabilized by C—H···π intermolecular interactions with a *Cg*···H distance of 3.7972 (14) Å.

The S–C, S==C and C–N bond distances are comparable to those of related structures (Fun *et al.* 2001; Raya *et al.* 2005).

### Experimental

A mixture of 6.44 ml of ethylaniline and 15.00 ml of concentrated aqueous ammonia in ice was added into 3.00 ml of ice-cold carbon disulfide and the resultant solution stirred for 6–7 h. The solid product obtained was filtered and rinsed three times with ice cold ethanol three times. The yellowish white was recrystallized in water/methanol mixture to yield crystal suitable for X-ray crystallographic analysis.

### Crystal data

**Table:**

C_18_H_20_N_2_S_4_	*F*(000) = 824
*M**_r_* = 392.6	*D*_x_ = 1.32 Mg m^−^^3^
Monoclinic, *C*2/*c*	Mo *K*α radiation, λ = 0.71073 Å
Hall symbol: -C 2yc	Cell parameters from 20252 reflections
*a* = 15.1923 (2) Å	θ = 2.3–28.5°
*b* = 11.5954 (2) Å	µ = 0.48 mm^−^^1^
*c* = 12.3762 (2) Å	*T* = 100 K
β = 115.039 (1)°	Block, yellow
*V* = 1975.31 (5) Å^3^	0.4 × 0.37 × 0.15 mm
*Z* = 4	

### Data collection

**Table:**

Bruker APEXII CCD diffractometer	2405 reflections with *I* > 2σ(*I*)
Graphite monochromator	*R*_int_ = 0.027
φ and ω scans	θ_max_ = 28.4°, θ_min_ = 2.3°
Absorption correction: multi-scan (*SADABS*; Bruker, 2008)	*h* = −20→18
*T*_min_ = 0.830, *T*_max_ = 0.931	*k* = −15→15
19720 measured reflections	*l* = −16→16
2481 independent reflections	

### Refinement

**Table:**

Refinement on *F*^2^	Primary atom site location: structure-invariant direct methods
Least-squares matrix: full	Secondary atom site location: difference Fourier map
*R*[*F*^2^ > 2σ(*F*^2^)] = 0.024	Hydrogen site location: inferred from neighbouring sites
*wR*(*F*^2^) = 0.068	H-atom parameters constrained
*S* = 1.06	*w* = 1/[σ^2^(*F*_o_^2^) + (0.0364*P*)^2^ + 1.5002*P*] where *P* = (*F*_o_^2^ + 2*F*_c_^2^)/3
2481 reflections	(Δ/σ)_max_ = 0.003
110 parameters	Δρ_max_ = 0.40 e Å^−^^3^
0 restraints	Δρ_min_ = −0.29 e Å^−^^3^

### Special details

**Table:**

Experimental. Carbon-bound H-atoms were placed in calculated positions [C—H = 0.98 Å for Me H atoms, 0.99 Å for Methylene H atoms and 0.95 Å for aromatic H atoms; *U*_iso_(H) = 1.2*U*_eq_(C) and were included in the refinement in the riding model approximation.
Geometry. All e.s.d.'s (except the e.s.d. in the dihedral angle between two l.s. planes) are estimated using the full covariance matrix. The cell e.s.d.'s are taken into account individually in the estimation of e.s.d.'s in distances, angles and torsion angles; correlations between e.s.d.'s in cell parameters are only used when they are defined by crystal symmetry. An approximate (isotropic) treatment of cell e.s.d.'s is used for estimating e.s.d.'s involving l.s. planes.
Refinement. Refinement of *F*^2^ against ALL reflections. The weighted *R*-factor *wR* and goodness of fit *S* are based on *F*^2^, conventional *R*-factors *R* are based on *F*, with *F* set to zero for negative *F*^2^. The threshold expression of *F*^2^ > σ(*F*^2^) is used only for calculating *R*-factors(gt) *etc*. and is not relevant to the choice of reflections for refinement. *R*-factors based on *F*^2^ are statistically about twice as large as those based on *F*, and *R*- factors based on ALL data will be even larger. The following ALERTS were generated. Each ALERT has the format test-name_ALERT_alert-type_alert-level. PLAT912_ALERT_4_C Missing # of FCF Reflections Above STh/*L*= 0.600 18 PLAT153_ALERT_1_G The su's on the Cell Axes are Equal ···. 0.00020 A ng. PLAT960_ALERT_3_G Number of Intensities with *I*. LT. - 2*sig(*I*) ··· 1

### Fractional atomic coordinates and isotropic or equivalent isotropic displacement parameters (Å^2^)

**Table:**

	*x*	*y*	*z*	*U*_iso_*/*U*_eq_	
C1	0.20064 (7)	0.61036 (8)	0.15533 (8)	0.01472 (18)	
C2	0.19847 (7)	0.51377 (9)	0.08775 (9)	0.01904 (19)	
H2	0.143	0.4979	0.0157	0.023*	
C3	0.27850 (8)	0.44056 (9)	0.12688 (10)	0.0220 (2)	
H3	0.2781	0.3747	0.081	0.026*	
C4	0.35898 (8)	0.46347 (9)	0.23282 (10)	0.0204 (2)	
H4	0.4137	0.4136	0.259	0.025*	
C5	0.35949 (8)	0.55942 (9)	0.30067 (9)	0.0201 (2)	
H5	0.4141	0.5739	0.3739	0.024*	
C6	0.28059 (7)	0.63409 (9)	0.26191 (9)	0.01764 (19)	
H6	0.2812	0.7003	0.3075	0.021*	
C7	0.12241 (7)	0.78155 (9)	0.03173 (9)	0.01789 (19)	
H7A	0.1905	0.8075	0.0581	0.021*	
H7B	0.0839	0.8481	0.0378	0.021*	
C8	0.08306 (9)	0.74308 (10)	−0.09751 (10)	0.0250 (2)	
H8A	0.1227	0.6795	−0.1047	0.038*	
H8B	0.0853	0.8078	−0.1472	0.038*	
H8C	0.0157	0.7171	−0.1241	0.038*	
C9	0.04038 (7)	0.67095 (8)	0.13196 (8)	0.01398 (18)	
N1	0.11909 (6)	0.68889 (7)	0.11179 (7)	0.01472 (16)	
S1	0.060555 (17)	0.55219 (2)	0.23600 (2)	0.01695 (8)	
S2	−0.060995 (17)	0.74667 (2)	0.07505 (2)	0.01764 (8)	

### Atomic displacement parameters (Å^2^)

**Table:**

	*U*^11^	*U*^22^	*U*^33^	*U*^12^	*U*^13^	*U*^23^
C1	0.0137 (4)	0.0164 (4)	0.0163 (4)	0.0020 (3)	0.0085 (3)	0.0022 (3)
C2	0.0173 (4)	0.0203 (5)	0.0180 (4)	0.0012 (4)	0.0059 (4)	−0.0024 (4)
C3	0.0231 (5)	0.0197 (5)	0.0247 (5)	0.0045 (4)	0.0116 (4)	−0.0020 (4)
C4	0.0171 (4)	0.0211 (5)	0.0250 (5)	0.0055 (4)	0.0107 (4)	0.0056 (4)
C5	0.0163 (5)	0.0234 (5)	0.0180 (4)	0.0005 (4)	0.0048 (4)	0.0032 (4)
C6	0.0186 (4)	0.0184 (4)	0.0164 (4)	−0.0001 (4)	0.0078 (4)	−0.0005 (3)
C7	0.0202 (5)	0.0167 (4)	0.0188 (4)	0.0004 (4)	0.0102 (4)	0.0032 (4)
C8	0.0332 (6)	0.0274 (5)	0.0183 (5)	0.0015 (4)	0.0146 (5)	0.0031 (4)
C9	0.0152 (4)	0.0147 (4)	0.0121 (4)	0.0013 (3)	0.0058 (3)	−0.0007 (3)
N1	0.0151 (4)	0.0154 (4)	0.0150 (4)	0.0025 (3)	0.0075 (3)	0.0020 (3)
S1	0.01842 (13)	0.01685 (13)	0.01994 (13)	0.00345 (8)	0.01235 (10)	0.00384 (8)
S2	0.01423 (13)	0.02036 (14)	0.01803 (13)	0.00421 (8)	0.00654 (10)	0.00127 (8)

### Geometric parameters (Å, º)

**Table:**

C1—C2	1.3898 (13)	C7—N1	1.4768 (12)
C1—C6	1.3910 (13)	C7—C8	1.5183 (15)
C1—N1	1.4454 (12)	C7—H7A	0.99
C2—C3	1.3910 (14)	C7—H7B	0.99
C2—H2	0.95	C8—H8A	0.98
C3—C4	1.3884 (15)	C8—H8B	0.98
C3—H3	0.95	C8—H8C	0.98
C4—C5	1.3920 (15)	C9—N1	1.3372 (12)
C4—H4	0.95	C9—S2	1.6495 (9)
C5—C6	1.3892 (14)	C9—S1	1.8205 (9)
C5—H5	0.95	S1—S1^i^	2.0112 (5)
C6—H6	0.95		
			
C2—C1—C6	121.25 (9)	N1—C7—H7A	109.1
C2—C1—N1	119.01 (8)	C8—C7—H7A	109.1
C6—C1—N1	119.71 (9)	N1—C7—H7B	109.1
C1—C2—C3	119.19 (9)	C8—C7—H7B	109.1
C1—C2—H2	120.4	H7A—C7—H7B	107.8
C3—C2—H2	120.4	C7—C8—H8A	109.5
C4—C3—C2	120.17 (10)	C7—C8—H8B	109.5
C4—C3—H3	119.9	H8A—C8—H8B	109.5
C2—C3—H3	119.9	C7—C8—H8C	109.5
C3—C4—C5	120.03 (9)	H8A—C8—H8C	109.5
C3—C4—H4	120	H8B—C8—H8C	109.5
C5—C4—H4	120	N1—C9—S2	125.78 (7)
C6—C5—C4	120.41 (9)	N1—C9—S1	110.70 (7)
C6—C5—H5	119.8	S2—C9—S1	123.49 (6)
C4—C5—H5	119.8	C9—N1—C1	121.70 (8)
C5—C6—C1	118.93 (9)	C9—N1—C7	121.70 (8)
C5—C6—H6	120.5	C1—N1—C7	116.18 (8)
C1—C6—H6	120.5	C9—S1—S1^i^	103.26 (3)
N1—C7—C8	112.52 (8)		
			
C6—C1—C2—C3	−0.94 (15)	S2—C9—N1—C7	−0.83 (13)
N1—C1—C2—C3	177.36 (9)	S1—C9—N1—C7	−179.17 (7)
C1—C2—C3—C4	0.63 (16)	C2—C1—N1—C9	85.13 (12)
C2—C3—C4—C5	0.48 (16)	C6—C1—N1—C9	−96.55 (11)
C3—C4—C5—C6	−1.31 (16)	C2—C1—N1—C7	−87.62 (11)
C4—C5—C6—C1	1.01 (15)	C6—C1—N1—C7	90.70 (11)
C2—C1—C6—C5	0.12 (15)	C8—C7—N1—C9	−87.47 (11)
N1—C1—C6—C5	−178.16 (9)	C8—C7—N1—C1	85.28 (11)
S2—C9—N1—C1	−173.18 (7)	N1—C9—S1—S1^i^	174.72 (6)
S1—C9—N1—C1	8.48 (11)	S2—C9—S1—S1^i^	−3.66 (7)

Symmetry code: (i) −*x*, *y*, −*z*+1/2.

### Hydrogen-bond geometry (Å, º)

Cg is the centroid of the C1–C6 ring.

**Table:**

*D*—H···*A*	*D*—H	H···*A*	*D*···*A*	*D*—H···*A*
C8—H8*B*···*Cg*^ii^	0.98	2.97	3.7972 (14)	143

Symmetry code: (ii) −*x*+1/2, −*y*+3/2, −*z*.

## References

[bb1] Bruker (2008). *APEX2*, *SAINT-Plus* and *SADABS* Bruker AXS Inc., Madison, Wisconsin, USA.

[bb2] Chieh, C. (1977). *Can. J. Chem.* **55**, 1116–1119.

[bb3] Farrugia, L. J. (1997). *J. Appl. Cryst.* **30**, 565.

[bb4] Farrugia, L. J. (1999). *J. Appl. Cryst.* **32**, 837–838.

[bb5] Fun, H.-K., Chantrapromma, S., Razak, I. A., Bei, F.-L., Jian, F.-F., Yang, X.-J., Lu, L. & Wang, X. (2001). *Acta Cryst.* E**57**, o717–o718. 10.1107/s0108270101005534 11408681

[bb6] McCleverty, J. A. & Morrison, N. (1976). *J. Chem. Soc. Dalton Trans.* pp. 2169–2175.

[bb7] Raya, I., Baba, I., Rosli, F. Z. & Yamin, B. M. (2005). *Acta Cryst.* E**61**, o3131–o3132.

[bb8] Sheldrick, G. M. (2008). *Acta Cryst.* A**64**, 112–122. 10.1107/S0108767307043930 18156677

[bb9] Victoriano, L. I. (2000). *Coord. Chem. Rev.* **196**, 383–398.

